# The effect of labiaplasty on sexual function, body dysmorphic symptoms, body ımage, self-esteem, and life satisfaction

**DOI:** 10.1590/1806-9282.20251079

**Published:** 2026-04-20

**Authors:** Yeliz Çeçen Dönmez, Esra Keles, Meziha Tasyurek, Sahra Sultan Kara, İsmail Bağlar, Cansu Ergenç

**Affiliations:** 1 University of Health Sciences, Kartal Dr. Lütfi Kırdar City Hospital, Department of Obstetrics and Gynecology – Istanbul, Turkey.; 2 University of Health Sciences, Kartal Dr. Lütfi Kırdar City Hospital, Department of Gynecologic Oncology – Istanbul, Turkey.; 3 University of Health Sciences, Hamidiye Faculty of Medicine, Department of Public Health – Istanbul, Turkey.; 4 Ankara Yildirim Beyazit University, Business School, Department of Finance and Banking – Ankara, Turkey.

**Keywords:** Sexual dysfunction, Body image, Self esteem, Body dysmorphic disorder

## Abstract

**OBJECTIVE:**

The aim of this study was to prospectively assess the effects of labiaplasty on female sexual function, genital self-image, body dysmorphic symptoms, self-esteem, and life satisfaction, and to examine the sociodemographic and motivational profiles of women undergoing the procedure.

**METHODS:**

A prospective single-center study was conducted between October 2024 and March 2025 on heterosexual, sexually active women undergoing labiaplasty. Women completed the Female Sexual Function Index, Female Genital Self-Image Scale, Rosenberg Self-Esteem Scale, Yale-Brown Obsessive Compulsive Scale modified for Body Dysmorphic Disorder, and Satisfaction With Life Scale before surgery and 3 months postoperatively.

**RESULTS:**

The study included 42 women with a mean age of 39.7±9.2 years. Postoperative assessments revealed significant improvements in genital self-image (p<0.001, Cohen's d=0.66), sexual function (p<0.001, d=0.67), and body dysmorphic symptoms (p=0.016, adjusted p=0.048, d=0.39). No significant changes were observed in self-esteem (p=0.857) or life satisfaction (p=0.071). A majority (69.0%) reported that others, predominantly family members, were the primary decision-makers in their relationships. Motivations included boosting self-confidence (22.0%), feeling "normal" (18.3%), and increasing sexual pleasure (11.9%), with participants endorsing multiple reasons (mean: 3.2±1.4). Most women had considered surgery for more than 1 year (73.9%).

**CONCLUSION:**

Labiaplasty significantly improves genital-specific outcomes but does not alter global psychological measures. The high prevalence of external influence on surgical decision-making highlights the need for comprehensive psychosocial assessment and patient-centered counseling prior to female genital cosmetic surgery.

## INTRODUCTION

Labiaplasty, involving restructuring of the labia minora and/or labia majora, has experienced a notable global increase over the past decade, attributed to growing acceptance of female genital cosmetic surgery (FGCS), and combined functional-aesthetic motivations^1^. Women seeking this surgery often cite physical discomfort, irritation from clothing, hygiene difficulties, and genital appearance dissatisfaction^2^. The appearance of external genitalia influences women's self-perception, sexual well-being, and quality of life^3,4^.

Previous studies examining psychological outcomes of labiaplasty remain limited by retrospective designs, small sample sizes, and a lack of standardized psychometric assessments^5^. Few prospective studies evaluate comprehensive psychosocial outcomes, including sexual function, genital self-image, body dysmorphic symptoms, self-esteem, and life satisfaction. Understanding how these interrelated domains are affected could enhance clinical decision-making and patient counseling^6^.

Despite the rising prevalence, significant research gaps persist. The literature lacks prospective studies that employs comprehensive validated psychometric assessments across multiple psychological domains, as well as detailed sociodemographic characterization of labiaplasty populations, particularly concerning decision-making autonomy and external influences. Evidence also limited regarding the relationships between motivational profiles and postoperative outcomes and contextualization within cultural frameworks where collectivist family dynamics influence surgical decision-making.

This study addresses these through prospective assessment of sexual function, genital self-image, body dysmorphic symptoms, self-esteem, and life satisfaction in women undergoing labiaplasty, while characterizing sociodemographic profiles, motivational factors, and decision-making dynamics within a Turkish clinical population.

## METHODS

This prospective single-center study was executed in the Department of Obstetrics and Gynecology, Kartal Dr. Lütfi Kırdar City Hospital between October 1, 2024, and March 1, 2025. Women undergoing labiaplasty were evaluated preoperatively and at 3 months postoperatively using validated questionnaires.

### Sample size calculation

G*Power analysis determined a minimum of 32 participants required for a moderate effect size (d=0.5), 80% power, α=0.05. The study enrolled 42 participants, exceeding minimum requirements while acknowledging limitations for subgroup analyses.

### Participants

Inclusion criteria were being a heterosexual, sexually active woman aged 18–65 years undergoing linear labiaplasty. Exclusion criteria included pregnancy, psychiatric illness, menopause, malignancy, previous vulvar or vaginal surgery, sexual inactivity, and alternative surgical techniques.

The study was approved by the institutional review board (No. 2024-15-873; 03/09/2024) and conducted in accordance with the Declaration of Helsinki, with written informed consent obtained from all participants.

### Measures

Participants completed a demographic data form and the following validated psychometric instruments.

### Female Genital Self-Image Scale

A seven-item instrument assesses women's perceptions of their genitalia. Items are rated on a four-point Likert scale (strongly agree to strongly disagree). Summed scores range from 7 to 28, where higher totals correspond to enhanced genital self-perception. The Turkish validation by Karadeniz^7^ demonstarated Cronbach's alpha of 0.90.

### Female Sexual Function Index

The scale assesses sexual function across six domains (desire, arousal, lubrication, orgasm, satisfaction, and pain) over the preceding 4 weeks. Turkish validity was established by Aygin and Aslan^8^. Scores span 2–36, values below 26 indicate sexual dysfunction.

### Rosenberg Self-Esteem Scale

This scale is a widely accepted instrument for assessing global self-esteem, validated in Turkish by Çuhadaroğlu^9^. Rosenberg conceptualized self-esteem as a global evaluation of the self, emphasizing a holistic and integrative approach to self-assessment. It consists of 10 items—five positively worded (items 1, 2, 4, 6, and 7) and five negatively worded (items 3, 5, 8, 9, and 10)—rated on a four-point Likert scale. Lower total scores indicate higher self-esteem.

### Satisfaction With Life Scale

A five-item instrument evaluating general life satisfaction^10^. Demonstrates robust reliability (α=0.88, test–retest r=0.97).

### Yale-Brown Obsessive Compulsive Scale—Body Dysmorphic Disorder version

It is a 12-item scale assessing BDD symptom severity (range: 0–40). Turkish adaptation by Altan^11^ demonstrated α=0.87. The Y-BOCS-BDD is the gold-standard instrument for quantifying appearance-related preoccupations and associated compulsive behaviors in cosmetic surgery populations, with excellent psychometric properties (α=0.80–0.89, intraclass correlation coefficients [ICCs]=0.88–0.99).

### Motivations assessment

Participants completed a demographic data form assessing sociodemographic characteristics, relationship patterns, cosmetic surgery history, procedures performed, and surgical motivations. Participants are allowed to select multiple options.

### Statistical analysis

Analyses were conducted using R (v.4.4.2). Normality was assessed via Kolmogorov-Smirnov tests with Lilliefors correction, Q-Q plots, and skewness/kurtosis values. All distributions demonstrated p>0.05 for Kolmogorov-Smirnov tests; skewness ranged from −0.87 to 1.24 (all |skewness|<3) and kurtosis from −1.12 to 2.45 (all |kurtosis|<10). Paired-samples t-tests evaluated pre-post changes on five psychometric outcomes. To address Type I error inflation from multiple comparisons, we applied the Holm-Bonferroni sequential correction method to maintain family-wise error rate at α=0.05. The Holm-Bonferroni method was selected over the traditional Bonferroni correction as it provides greater statistical power while controlling for multiple comparisons. Both unadjusted and adjusted p-values are reported. Cohen's d effect sizes were calculated (small=0.2, medium=0.5, large=0.8). Significance was set at p<0.05.

## RESULTS

### Participant characteristics

Forty-two women underwent labiaplasty (mean age: 39.7±9.2 years; body mass index: 26.7±5.9). Most were married (83.3%), and 66.7% reported sexual satisfaction. Smoking was reported by 38.1%. The mean duration of the relationship was 17.8 ± 11.9 months. Regarding relationship decision-making, 2.4% reported independent decisions, 14.3% identified husbands as primary decision-makers, 14.3% reported joint decision-making, and 69.0% indicated that others, predominantly family members, were primary decision-makers (Table 1).

**Table 1:** Demographic and clinical characteristics of the participants (n=42).

Variable	Categories	Mean±SD/n (%)
Age (years)	–	39.7±9.2
Marital status	Married	35 (83.3)
	Single	4 (9.5)
	In a relationship	1 (2.4)
	Divorced	1 (2.4)
	Widowed	1 (2.4)
Educational attainment	No formal education	5 (11.9)
	Primary school	10 (23.8)
	Secondary school	8 (19.0)
	High school	14 (33.3)
	University degree	5 (11.9)
BMI	–	26.7±5.9
Smoking status	Smoker	16 (38.1)
	Non-smoker	26 (61.9)
Relationship duration (months)	–	17.8±11.9
Duration of longest relationship (months)	–	19.2±11.2
Operation duration (minutes)	–	27.5±14.6
Primary decision-maker in the relationship	Self	1 (2.4)
	My husband	6 (14.3)
	Joint decision-making	6 (14.3)
	Others (family members)	29 (69.0)
Sexual satisfaction	Satisfied	28 (66.7)
	Not satisfied	14 (33.3)
Partner's educational attainment	No formal education	4 (9.5)
	Primary school	5 (11.9)
	Secondary school	2 (4.8)
	High school	16 (38.1)
	University degree	11 (26.2)
	Master's degree	4 (9.5)
Type of anesthesia administered	General anesthesia	42 (100.0)
Postoperative complications	Absent	42 (100.0)

Data are expressed as mean±SD or number (percentage) where appropriate. SD: standard deviation; BMI: body mass index.

Duration of surgical consideration: 42.9% contemplated procedure >3 years, 31.0% for 1–3 years, 14.3% for 3–6 months, and 11.9% for 6–12 months. Prior cosmetic procedures: 9.5% Botox, 9.5% breast implants, and 4.8% rhinoplasty; 78.6% had no prior procedures. The most commonly performed surgeries were labiaplasty of the labia majora (83.3%), labia minora (42.9%), and hoodoplasty (9.3%).

### Motivational profile

Participants endorsed multiple motivations (mean: 3.2±1.4, range: 1–7). Motivations for surgery included boost self-confidence (22.0%), feel "normal" (18.3%), improve aesthetic appearance (10.1%), eliminate discomfort (11.9%), increase sexual pleasure (11.9%), enhanced sensation (9.2%), satisfy partner (10.1%), partner insistence (4.6%), and other (1.8%), respectively (Table 2).

**Table 2:** Patient history, procedure type, and motivations for female genital cosmetic surgery (n=42).

Variable	Categories	Mean±SD/n (%)
How long have you been considering undergoing this cosmetic surgery?	3–6 months	6 (14.3)
	6–12 months	5 (11.9)
	1–3 years	13 (31.0)
	More than 3 years	18 (42.9)
Have you previously undergone any cosmetic surgery?*	None	33 (78.6)
	Botox	4 (9.5)
	Rhinoplasty	2 (4.8)
	Breast implants	4 (9.5)
	Other	2 (4.8)
Procedure performed*	Labiaplasty, labia minora	18 (42.9)
	Labiaplasty, labia majora	35 (83.3)
	Perineoplasty	0
	Hoodoplasty	4 (9.3)
My primary reason(s) for undergoing the surgery were: (n=42)*	To look better "down there"	11 (10.1)
	To feel more "normal"	20 (18.3)
	To boost my self-confidence	24 (22.0)
	To eliminate discomfort from clothing, sexual, or sports activities	13 (11.9)
	To experience enhanced sensation during sex	10 (9.2)
	To increase my sexual pleasure	13 (11.9)
	To satisfy my sexual partner	11 (10.1)
	Mainly due to my partner's insistence	5 (4.6)
	Other	2 (1.8)

Data are expressed as mean±SD or number (percentage) where appropriate.

* Participants were permitted to select multiple options. SD: standard deviation.

### Psychometric outcomes

Postoperatively, Y-BOCS-BDD scores improved significantly (t=-2.52, p=0.016, p_adjusted=0.048, 95%CI −0.21, −0.02, d=0.39). FSFI scores improved substantially (t=4.32, p<0.001, p_adjusted<0.001, 95%CI 0.19, 0.54, d=0.67). FGSIS scores decreased significantly (t=-4.25, p<0.001, p_adjusted<0.001, 95%CI −0.53, −0.19, d=0.66), indicating improved genital self-image. No significant changes occurred in RSES (t=-0.18, p=0.857, p_adjusted=0.857, 95%CI −0.09, 0.08) or SWLS (t=-1.86, p=0.071, p_adjusted=0.142, 95%CI −0.38, 0.02) (Table 3).

**Table 3:** Pre- and postoperative psychometric outcomes (n=42).

Scale	Mean Diff.	SD	t	p (unadj)	p (Holm)	95%CI (lower, upper)	Cohen's d
Y-BOCS-BDD	-0.119	0.31	-2.52	0.016	**0.048**	[-0.21, −0.02]	0.39
FSFI	0.37	0.55	4.32	<0.001	**0.001**	[0.19, 0.54]	0.67
FGSIS	-0.36	0.55	-4.25	<0.001	**<0.001**	[-0.53, −0.19]	0.66
RSES	-0.008	0.28	-0.18	0.857	>**0.857**	[-0.09, 0.08]	-
SWLS	-0.18	0.63	-1.86	0.071	>**0.142**	[-0.38, 0.02]	-

Y-BOCS-BDD: Yale-Brown Obsessive Compulsive Scale-Body Dysmorphic Disorder; FSFI: Female Sexual Function Index; FGSIS: Female Genital Self-Image Scale; RSES: Rosenberg Self-Esteem Scale; SWLS: Satisfaction With Life Scale; SD: standard deviation; CI: confidence interval. Holm-Bonferroni sequential correction applied to control family-wise error rate. Bold values indicate statistically significant results (p<0.05).

## DISCUSSION

This prospective study demonstrated that labiaplasty significantly improves genital-specific outcomes, including sexual function, body dysmorphic symptoms, and genital self-image but does not alter global psychological measures including self-esteem and life satisfaction. These findings align with prior research showing localized improvements without broader psychological transformation^12^.

The significant improvements in genital-specific outcomes, without changes in global measures suggests that labiaplasty effectively addresses the targeted concerns related to genital appearance and function, while not act as a panacea for broader well-being. Life satisfaction is influenced by a broader array of psychosocial and environmental factors extending beyond genital appearance^13^. Global self-esteem, conceptualized as holistic self-evaluation, similarly resists modification through localized anatomical interventions^14^.

The substantial improvement in sexual function reflects meaningful enhancement across desire, arousal, lubrication, orgasm, satisfaction, and pain domains, aligning with prior research^6^. The reduction in body dysmorphic symptoms indicates that appearance-related preoccupations and compulsive behaviors diminish when anatomical concerns are surgically addressed.

Motivations were predominantly psychosexual, with self-confidence enhancement (22.0%) and feeling "normal" (18.3%) as primary drivers, consistent with previous research^15^. The desire to feel "normal" is particularly resonant, given the pervasive societal emphasis on idealized genital aesthetics fueled by media^16–18^, which can lead to a belief that one's own anatomy is "abnormal"^19^. The extended period of consideration for the procedure indicates that the decisions were not impulsive, but reflected long-standing concerns. However, 14.7% cited partner-oriented motivations (satisfaction and insistence), raising questions about external influence.

Another significant finding concerns limited decision-making autonomy, with 69.0% reporting that others, such as family members as the primary relationship decision-makers and only 2.4% reporting autonomous decision-making. This pattern demands contextualization within Turkish sociocultural frameworks. Turkey's intersection of European and Middle Eastern influences creates complex negotiations between modernization and traditional values. Family-centered decision-making remains prevalent, particularly for matters affecting family honor or marital harmony. In collectivist cultural frameworks, predominant across Middle Eastern, Asian, and Mediterranean societies, individual autonomy is often subordinated to family collective welfare, contrasting with Western biomedical ethics prioritizing individual patient autonomy^20^.

These findings impose that clinicians must provide enhanced preoperative psychological assessment exploring decision-making dynamics and external influences, hold private patient consultations separate from family members, demonstrate cultural competence by navigating tensions between respecting cultural differences and identifying coercion, explore expectations in detail, conceptualize informed consent as ongoing dialog rather than a discrete event, and refer patients exhibiting external pressure or ambivalence to psychological support. Clinicians must distinguish between culturally normative family involvement and explicit coercion undermining individual agency. While avoiding imposition of Western individualistic autonomy notions, clinicians must remain vigilant where external pressure undermines authentic agency^21,22^.

### Limitations

Several limitations warrant acknowledgment. First, the single-center design and small sample size may restrict the generalizability of the findings. Multicenter studies with larger samples are needed. Second, the study lacks long-term follow-up, thus, cannot establish outcome durability, late complications or surgical regret. Extended follow-up with comprehensive complication surveillance remains essential. Third, restriction to linear resection limits applicability to alternative techniques, Fourth, autonomy assessment via single self-report item may not capture nuanced influence dynamics; sophisticated multi-item instruments and qualitative methodologies would provide richer understanding. Despite these constraints, this study represents one of the more detailed examinations of sociodemographic factors and the nuanced motivations for labiaplasty within a specific cohort, providing valuable insights into decision-making dynamics, particularly the role of external influence.

## CONCLUSION

Labiaplasty significantly improved body dysmorphic symptoms, sexual function, and genital self-image without altering overall self-esteem or life satisfaction. The limited decision-making autonomy underscores the need for comprehensive psychosocial assessment and culturally sensitive counseling prior to FGCS. Careful patient selection, realistic expectation management, and attention to external pressures remain paramount. Future research should employ multicenter longitudinal designs with qualitative components to assess long-term outcomes, decision-making factors, and media influence on genital self-perception.

## Data Availability

The datasets generated and/or analyzed during the current study are available from the corresponding author upon reasonable request.
